# Loneliness and Social Isolation in Individuals with Acute Myocardial Infarction and Takotsubo Syndrome: A Scoping Review

**DOI:** 10.3390/healthcare13060610

**Published:** 2025-03-12

**Authors:** Gabriele Lo Buglio, Gianluca Cruciani, Marianna Liotti, Federica Galli, Vittorio Lingiardi, Annalisa Tanzilli

**Affiliations:** 1 Department of Dynamic and Clinical Psychology and Health Studies, Sapienza University of Rome, Via degli Apuli, 1, 00185 Rome, Italy; gabriele.lobuglio@uniroma1.it (G.L.B.); marianna.liotti@uniroma1.it (M.L.); f.galli@uniroma1.it (F.G.); 2 Department of Systems Medicine, University of Rome Tor Vergata, Via Montpellier, 1, 00133 Rome, Italy; gianluca.cruciani@uniroma2.it; 3 Department of Biology and Biotechnologies “Charles Darwin”, Sapienza University of Rome, Piazzale Aldo Moro, 5, 00185 Rome, Italy

**Keywords:** myocardial infarction, Takotsubo syndrome, loneliness, social isolation, scoping review

## Abstract

Background/Objectives: Loneliness and social isolation are considered significant social determinants of myocardial infarction (MI) or Takotsubo syndrome (TS). However, research on these factors in MI populations is highly heterogeneous, and evidence regarding TS is sparse. The present scoping review aimed at mapping the extent and breadth of the literature on loneliness and social isolation in individuals with MI or TS. Methods: Following PRISMA-ScR guidelines and JBI methodology, we conducted a systematic search in PubMed, Web of Science, and EBSCO/PsycINFO, supplemented by a manual search, for studies published up to 25 June 2024. Primary research studies on loneliness and/or social isolation in individuals with MI or TS were included based on predefined inclusion and exclusion criteria, with title–abstract and full-text screening. Results: Sixteen studies met the inclusion criteria, all of which focused on MI. Studies were categorized into three key concepts: loneliness (*k* = 5), social isolation (*k* = 7), and both loneliness and social isolation (*k* = 4). The findings showed that MI impacts subjective experiences and interpersonal relationships, often leading to identity shifts and increased social isolation and loneliness. These factors have been shown to be associated with severe physical health outcomes, including heightened mortality risk; however, these associations appear to be highly mediated by unhealthy lifestyle behaviors. Notably, no studies on TS met the inclusion criteria, highlighting a significant research gap. Additionally, no study explored clinical interventions targeting social isolation or loneliness. Conclusions: MI has a profound impact on social and emotional well-being, with loneliness and social isolation contributing to severe health outcomes. Further research is needed to understand the impact of these factors on individuals with TS.

## 1. Introduction

Cardiovascular diseases (CVDs) are the leading cause of mortality worldwide, accounting for an estimated 17.9 million deaths annually [1]. A recent global review estimated that, in 2020, acute coronary syndromes (ACSs) contributed to 18–23% of all CVD-related deaths [2]. ACSs encompass a group of clinical syndromes characterized by acute myocardial ischemia, defined as a sudden reduction in blood supply to the heart. Common symptoms include chest pain, dyspnea, electrocardiographic abnormalities, and loss of vitality of cardiac segments [3,4]

Among ACS, myocardial infarction (MI) is recognized as the most severe clinical presentation, as it represents the leading cause of human death worldwide [5]. The diagnosis of MI necessitates a spike and/or decline in cardiac troponin with at least a single reading above the upper reference limit of the 99th percentile and any of the following: symptoms of myocardial ischemia, new ischemic changes on the electrocardiogram, imaging evidence of new loss of viable myocardium, or the identification of thrombosis on coronary angiography [6]. MI could be further classified following changes on the electrocardiogram into ST-segment elevation myocardial infarction (STEMI) and non-ST-segment elevation myocardial infarction (NSTEMI), also reflecting the degree of reduction in blood supply [3]. The global prevalence of MI is approximately 9.5% in individuals over 60 and 3.8% in those below 60 [5]. MI-related hospitalization occurs more frequently in men than women. However, women who are hospitalized are characterized by older age, a higher prevalence of risk factors such as diabetes, hypertension, and depression, and more negative outcomes, including higher mortality rates [7].

Takotsubo syndrome (TS), also known as broken heart syndrome, apical ballooning syndrome, or stress cardiomyopathy, is another ACS that has been garnering increasing clinical attention over the past decade. TS manifests as transient heart failure that mimics MI but occurs without coronary artery obstruction and is typically associated with a favorable prognosis. While its precise etiology remains unclear, proposed pathophysiological mechanisms include catecholamine surges, metabolic dysfunction, hormonal imbalances, and inflammatory responses [8,9]. TS accounts for approximately 1–3% of ACS cases, with prevalence reaching 5–6% among postmenopausal women [10,11]. Notably, approximately 80% of TS patients report having experienced a major stressful event preceding the acute cardiac episode, highlighting the critical role played by psychological factors in its onset and progression [12,13,14]. When triggered by emotional stressors, TS is typically associated with a favorable prognosis and complete recovery of left ventricular (LV) contractility [15]. Conversely, TS precipitated by a physical trigger may lead to immediate myocardial injury and the activation of cell survival pathways, potentially increasing the risk of chronic LV dysfunction and adverse long-term outcomes [16]. These findings suggest that TS results from a complex interplay of structural, physiological, and psychological factors.

Preventing ACS by addressing risk factors is essential for improving both overall quality of life and life expectancy. Effective prevention not only reduces the risk of initial and recurrent cardiac events but also minimizes the likelihood of long-term complications, such as heart failure and arrhythmia. Additionally, it offers significant economic benefits by decreasing hospital admissions, emergency care costs, and the need for complex and expensive medical procedures [17]. While traditional modifiable risk factors (e.g., hypertension, hyperlipidemia, diabetes, smoking) can be managed through targeted interventions to significantly reduce ACS incidence [18], increasing attention has been directed toward social determinants of health, which are now recognized as critical intervention targets to prevent the exacerbation of various health conditions [19,20]. Among these social determinants, social isolation and loneliness have been identified as significant risk factors for adverse health outcomes, including increased mortality, especially among older adults [21,22].

It is important to distinguish between these two constructs, as they may contribute to negative health outcomes through distinct mechanisms and require different intervention strategies [23]. Social isolation is an objective measure of an individual’s social connections, often quantified as the number of meaningful relationships in their social network [24]. Loneliness, on the other hand, is a subjective feeling that arises from a perceived discrepancy between one’s desired and actual social relationships. Thus, individuals may feel lonely despite having social connections, or vice versa [25].

Both social isolation (objective) and loneliness (subjective) have been consistently linked to negative health outcomes [21,26,27], including increased risk of CVD [28,29]. Given the significant impact of socio-psychological factors on ACS development and progression, it is crucial to understand the impacts of loneliness and social isolation on patients with MI and TS [30]. However, previous studies on MI have demonstrated high methodological heterogeneity, and the role played by these factors in TS remains poorly understood. This gap highlights the need for a comprehensive review to synthesize current knowledge. Given the heterogeneity of studies in the literature and in accordance with methodological guidelines [31], a scoping review was conducted to systematically map existing research, identifying the types of evidence available and research gaps. In more detail, the scoping review aimed to accomplish the following objectives:

1. Explore the subjective experiences and attitudes associated with loneliness/social isolation in individuals with MI or TS;
2. Examine the relationship between loneliness/social isolation and physical health and mental health outcomes;
3. Identify clinical strategies to address loneliness and social isolation in these populations.

## 2. Materials and Methods

### 2.1. Search Strategy and Selection Criteria

The study adhered to the PRISMA-ScR guidelines [32] and the JBI methodology for scoping reviews [33,34], and the protocol was prospectively registered: https://osf.io/q48td (registered on 17 July 2024). The PRISMA-ScR checklist is available in Appendix A, and any protocol amendments are reported in Appendix B. Ethics/Institutional Review Board approval was not required, as the study comprised a scoping review. The study design was also informed by prior research [35]. A preliminary search in EBSCO/PsycINFO and PubMed was conducted to identify relevant studies, informing the development of a detailed search strategy: ((“loneliness”) OR (“social isolation”) OR (“social alienation”) OR (“social withdrawal”) OR (“social distance”) OR (“interpersonal relations”) OR (“psychosocial deprivation”) OR (“lonely”) OR (“solitude”)) AND (((“acute coronary syndrome”) OR ((“myocardial infarction”) AND (“type”)) OR (“acute myocardial infarction”) OR (“Takotsubo”))). This search string was subsequently applied to PubMed, EBSCO/PsycINFO, and Web of Science, covering publications from inception to 25 June 2024. Additional articles were identified through manual searches. All retrieved citations were uploaded to Rayyan (https://www.rayyan.ai/ accessed on 17 July 2024) for duplicate removal. A pilot screening was conducted, in which two authors independently reviewed titles and abstracts against predefined inclusion criteria. Following this, all titles and abstracts were systematically screened, and full texts of potentially relevant studies were assessed for eligibility. Reasons for exclusion were documented. Any disagreements were resolved through consultation with additional authors. The entire search process, including the study selection, is documented and presented, along with the PRISMA-ScR flow diagram illustrating the selection process.

Studies were included if they examined individuals diagnosed with MI or TS (“population”), focused on loneliness/social isolation (“concept”), and were assessed through validated measures or focus groups/interviews. This also encompassed studies defining social isolation and/or loneliness using clearly defined criteria (regardless of whether a psychometric tool was employed), as well as empirically derived group classifications. No restrictions were applied based on context. Only primary research studies were included (“design”). Reviews and studies published in languages other than English or Italian were excluded. Additionally, studies that did not provide separate data for TS and/or MI were excluded.

### 2.2. Data Extraction

The first author conducted the data extraction, while other authors provided feedback. Extracted data included the country of study, sample characteristics, publication type, year of study, study design, research objectives, main findings, clinical diagnosis, measures used to assess loneliness/social isolation, details about patients’ subjective experiences of loneliness/social isolation, details about patients’ associated physical and mental health outcomes, and details about the interventions used to target loneliness/social isolation. The data extraction tool was iteratively refined throughout the data charting process.

### 2.3. Data Analysis and Presentation

Study designs, socio-demographic characteristics, research aims, and main results were systematically compiled into a summary table. Additionally, findings relevant to the core research questions of the present scoping review were analyzed and categorized into three thematic areas: “loneliness”, “social isolation”, and “both loneliness and social isolation”.

## 3. Results

### 3.1. Study Selection

**Figure 1:**
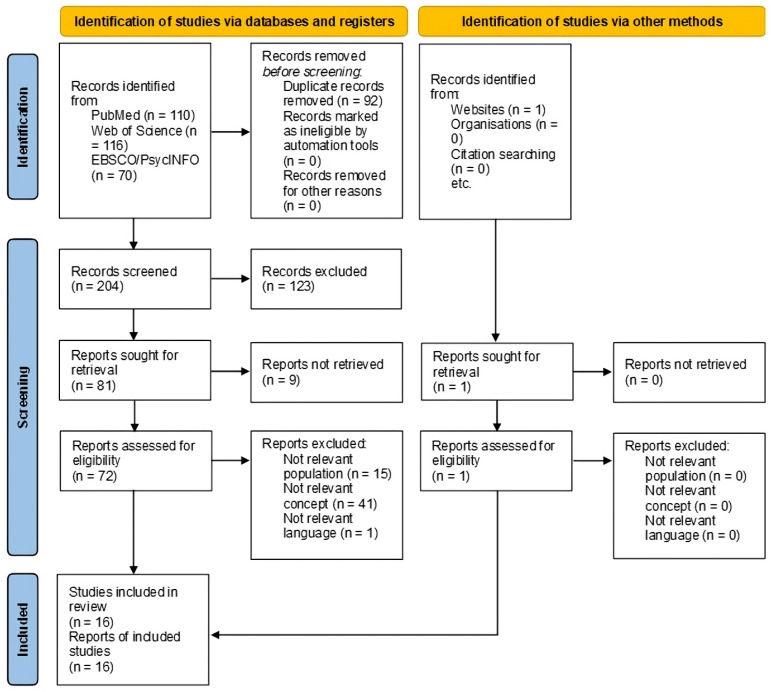
PRISMA-ScR flow diagram illustrating the literature search and selection process.

The search identified 296 reports, of which 204 remained after duplicate removal was assessed at the title–abstract level. Subsequently, 73 articles underwent full-text screening, and 16 studies met the inclusion criteria and were included in the scoping review [36,37,38,39,40,41,42,43,44,45,46,47,48,49,50,51]. Figure 1 presents the flowchart illustrating the study selection process, while Table 1 summarizes the main characteristics of the included studies. Among the selected studies, 6 had cross-sectional designs, while 10 employed longitudinal cohort designs. Geographically, 11 studies were conducted in Europe, 4 in North America, and 1 in Oceania. Publication years ranged from 1973 to 2023, distributed as follows: one in the 1970s, one in the 1980s, two in the 1990s, five in the 2000s, two in the 2010s, and five in the 2020s. Based on their primary focus, studies were categorized into three concepts: loneliness (*k* = 5), social isolation (*k* = 7), and both loneliness and social isolation (*k* = 4).

### 3.2. Loneliness

Five studies [46,47,48,49,50] were included in this concept, with three examining the subjective experience of loneliness and two assessing its impact on physical and mental health outcomes.

Among the studies exploring subjective experiences, two employed a qualitative approach, recruiting women who had survived MI and exploring their close relationships. Sundler et al. [46] highlighted that close relationships and sexuality—both of which may be profoundly affected by MI—were integral to women’s perceived well-being and quality of life. Their findings suggest that while MI often intensifies the need for meaningful companionship and intimacy (including sexual connection), pre-existing relational tensions may intensify feelings of loneliness, leading to worry, discomfort, anger, and isolation. Another study [47] interviewed both women who had experienced MI and their partners, revealing that post-MI experiences of overprotection and restricted autonomy frequently contributed to emotional distress. Moreover, both patients and their partners tended to withhold their feelings about the illness, further exacerbating patients’ experiences of loneliness. A separate research [48] compared psychosocial differences between MI patients and age-matched healthy controls, reporting significantly higher loneliness among the MI group.

Regarding health outcomes, Zuccarella-Hackl et al. [50] empirically identified three distinct clusters of MI patients: “lonely”, “low risk”, and “avoidant”. The “lonely” cluster was characterized by the lowest scores in resilience and social support and average scores in task-oriented coping and positive affect. Patients in this cluster also demonstrated higher levels of depressive and post-traumatic stress symptoms compared to the “low-risk” group. In line with these findings, Thompson and Watson [49] identified loneliness as the most significant factor negatively impacting the quality of life in MI patients.

### 3.3. Social Isolation

Seven studies were included in this concept [36,37,38,39,40,42,45], with two examining subjective experiences or attitudes related to social isolation and five assessing the impact of social isolation on physical health, mental health, and functional outcomes.

Among the studies exploring subjective experiences, a qualitative investigation by Dryer et al. [39] highlighted that MI profoundly disrupts patients’ sense of safety and security, leading to perceived identity shifts (i.e., becoming a “different person”). Following MI, patients reported significant losses and negative experiences, including social isolation, heightened vulnerability, uncertainty about the future, and difficulties in expressing emotions—often compounded by a pervasive sense of fear. However, some also reported greater emotional presence with both themselves and others post-MI. The authors emphasized the importance of addressing these emotional and psychological changes in the recovery process. In contrast, adopting a quantitative rather than a qualitative approach, Baigi et al. [36] compared MI patients who had declined cardiac rehabilitation with those who had undergone such treatment, exploring attitudes towards social isolation, the personal impact of social isolation, and the relevance of social isolation for coronary heart disease. The results showed no significant differences between groups.

Regarding physical health outcomes, Ruberman et al. [45] found that MI patients experiencing high social isolation and life stress faced a mortality risk of more than four times greater than those with lower levels of these factors. Partially supporting these findings, Cleophas et al. [37] reported that MI patients one year before, during, and one year after MI were more socially isolated than aged-matched controls in at least one of the following areas: “no talking” and “no visits”. Notably, “no talking” was independently associated with an increased risk of MI. In terms of mental health outcomes, Dickens et al. [38] identified several factors independently associated with persistent depressive disorder prior to MI, including social isolation, younger age, female gender, a previous psychiatric history, significant non-health-related stressors, and the absence of a close confidant. Moreover, patients who exhibited persistent depressive disorder at baseline and severe depressive symptoms at the 12-month follow-up were more likely to be female, have a history of psychiatric illness, be socially isolated, and experience ongoing social difficulties. However, social isolation did not predict worsening depressive symptoms in patients who were not already depressed at baseline. Focusing on the quality of life, Ecochard et al. [40] found that 29% of MI patients reported dissatisfaction in the domain of social isolation 1-year post-MI. Finally, Ickovics et al. [42] examined functional outcomes, revealing that social class—categorized as “highest”, “middle”, and “lowest”—significantly influenced recovery. Even after controlling for relevant clinical, demographic, and psychosocial factors (including social isolation), patients from the lowest and middle social classes had a reduced likelihood of functional improvement compared to those in the highest social class.

### 3.4. Both Loneliness and Social Isolation

Four studies were included in this concept [41,43,44,51]. One focused on MI survivors’ subjective experiences of loneliness and social isolation, while the other three utilized quantitative methods to explore the impact of these factors on physical health outcomes.

Liljeroos et al. [44] conducted a qualitative study investigating the emotional and behavioral adjustments of MI survivors at the outset of internet-based cognitive–behavioral therapy. The results showed that these individuals experienced significant increases in anxiety (especially heart-focused anxiety, characterized by excessive concerns related to cardiovascular health) and depression following MI, contributing to shifts in self-perception and identity. To cope with their emotional distress, patients often withdrew socially and avoided specific experiences, reinforcing their distress and social isolation.

In a quantitative study, Freak-Poli et al. [51] examined a sample of 11,486 community-dwelling individuals aged 70 years and older, finding that social isolation was associated with the onset of CVD, while loneliness was linked to fatal CVD (mean follow-up: 4.43 years). However, neither loneliness nor social isolation showed a significant association with incident MI. In a large-sampled study (N = 479,054) by Hakulinen et al. [41], both social isolation and loneliness were found to increase the risk of MI and stroke in the general population (mean follow-up: 7.1 years). Furthermore, social isolation (but not loneliness) predicted higher mortality rates in individuals with a history of MI or stroke. These associations were largely explained by other risk factors, such as unhealthy lifestyle behaviors, socio-economic status, and poor mental health. Partially aligning with these findings, Liang et al. [43] analyzed 19,360 patients with type 2 diabetes mellitus, finding that social isolation significantly increased the risk of all-cause and cardiovascular-related mortality (median follow-up: 12.4 years). In contrast, loneliness was associated with a higher risk of non-fatal MI or stroke. These associations were mediated by behavioral factors, including physical activity, smoking, sleep duration, alcohol consumption, TV watching time, and diet.

## 4. Discussion

The present scoping review mapped the extent and type of literature related to loneliness and social isolation in individuals with MI. Three key findings emerged. First, qualitative studies revealed that MI impacts subjective well-being, interpersonal connections, and social relationships, often leading to identity shifts and increased loneliness and social isolation. Second, quantitative studies highlighted that social isolation and loneliness are associated with adverse physical health outcomes, including increased risk of MI and/or mortality. However, these associations are largely mediated by risk factors such as unhealthy lifestyle behaviors. Third, a significant research gap was identified, as no studies addressed TS (despite its inclusion in the original protocol), and no research examined clinical interventions aimed at mitigating social isolation and loneliness in patients with MI or TS.

MI is a life-altering event that may reshape one’s perception of “normality”, necessitating adaptation to a new reality [59]. The present review highlighted that MI patients frequently experience heightened anxiety, depression, social isolation, and loneliness [44], which can exacerbate the disease burden and worsen prognosis. Moreover, interpersonal relationships may evolve over time, with some studies reporting both an increased desire for social connection and a deterioration of interpersonal relationships following MI [46,47]. Notably, some studies highlighted that social isolation may predate MI [37]. Given these psychosocial shifts, addressing the losses and gains associated with MI appears crucial for promoting personal recovery [39].

Several studies have linked social isolation and loneliness to poorer health outcomes and increased mortality risk [21,26,27]. In their Evolutionary Theory of Loneliness, Cacioppo and Cacioppo [60] postulated that perceived social isolation promotes short-term species survival by heightening alertness and implicit vigilance for social threats, increasing self-centeredness as a means of self-preservation and triggering a cascade of behavioral, neural, hormonal, cellular, and molecular responses aimed at repairing or replacing lost social connections. These responses include sleep disturbances, activation of the hypothalamic–pituitary–adrenocortical (HPA) axis, enhanced sympathetic tonus, altered leukocyte transcriptome dynamics, decreased viral immunity, increased inflammatory markers, greater prepotent responding, and higher depressive symptomatology—all of which have been empirically associated with poorer general health outcomes and increased mortality rates [60]. Sharma et al. [61] further proposed a model illustrating how loneliness and social isolation may contribute to adverse cardiovascular outcomes through similar mechanisms. Specifically, they highlighted that both conditions are associated with exaggerated autonomic stress responses and sympathetic nervous system hyperactivity, leading to increased total peripheral resistance, reduced heart rate variability, hypertension, ischemic heart disease, and diminished cardiac output. Additionally, they observed that social isolation over-activates the HPA axis, resulting in chronically elevated glucocorticoid levels, systemic inflammation, endothelial dysfunction, and platelet activation—all of which accelerate atherosclerosis. Nevertheless, loneliness and social isolation could be associated with MI also through psychological mechanisms, for example, attachment patterns. Insecure attachment has been linked to both enhanced loneliness and social isolation [62,63] and MI risk [64,65]. Given the stability of the behavioral and neurophysiological correlates associated with insecure and disorganized attachment, it is likely that these patterns persist throughout an individual’s development, sustaining disruptions in interpersonal functioning and, ultimately, contributing to both MI risk as well as loneliness and social isolation [66,67,68,69,70,71,72].

Loneliness and social isolation may also negatively impact cardiovascular health through behavioral pathways. Both conditions have been consistently linked to a wide range of risky behaviors across the lifespan, including physical inactivity, unhealthy dietary habits, smoking, and alcohol and substance use [73,74]. Notably, such behaviors are considered modifiable risk factors for ACS [3,4], and intervention programs targeting these factors have been shown to reduce negative health outcomes, rehospitalization risk, and mortality among MI patients [75,76]. Although the exact mechanisms linking loneliness and social isolation to risk behaviors remain unclear, several hypotheses have been proposed. For instance, it has been suggested that these conditions could reduce individuals’ perceived responsibility to maintain their health for the sake of loved ones, disconnect them from social norms that promote healthy behaviors, and deprive them of both emotional and instrumental social support that would encourage health-conscious decisions and behaviors [77]. Additionally, loneliness and social isolation have been associated with higher levels of depression and anxiety [78], which, in turn, have been linked to increased engagement in risky behaviors, such as smoking, alcohol consumption, and drug use—often employed as maladaptive coping strategies [79,80]. Such behaviors may exacerbate ACS risk. Notably, depression and anxiety are highly prevalent among individuals with MI [81,82] and have been linked to more adverse long-term outcomes, including increased mortality risk [83,84]. Moreover, given the significant life impact of MI, affected individuals frequently experience high levels of psychological distress [44].

Furthermore, the present findings support the hypothesis that social isolation and loneliness are independent predictors of adverse ACS-related outcomes [73]. This suggests that both the objective condition of social isolation and the subjective experience of loneliness may contribute to heightened health risks. In other words, feeling alone, rather than being alone, may be sufficient to worsen health outcomes. This underscores the importance of integrating patient-reported outcomes into clinical practice, as they may provide valuable insights extending beyond traditional clinical measures. “Patient-reported outcomes” may encompass a broad range of subjective patient assessments, referring to symptoms, functioning, well-being, treatment, quality of care, and clinical/professional communication [85,86]. Patients may convey these assessments through various formats, offering a comprehensive perspective on their condition, its impact, and its functional consequences. In the field of ACS, patient-reported outcomes have been associated with improved prediction of acute events [87] and long-term survival [88]. Moreover, by capturing critical aspects of patient health (e.g., psychological well-being, social functioning, overall life satisfaction), they facilitate the development of personalized treatment plans and enhance healthcare efficiency. As loneliness may be considered a subjective counterpart to social isolation, and in view of its association with health-related negative outcomes, future research should incorporate patient-reported outcomes to assess the trajectory of cardiac events within ACS populations.

Of note, none of the studies included in the present scoping review focused on TS. During the study selection process, some reports were excluded that, while addressing TS during the COVID-19 pandemic (e.g., [89,90]), did not meet the predefined inclusion criteria. Specifically, these studies did not measure loneliness or social isolation, nor did they conduct interviews to evaluate these factors. In this vein, the present scoping review fulfilled its objective of identifying gaps in the literature as avenues for further research [32,33,35]. Future studies should seek to employ validated measures or qualitative interviews to assess social isolation and loneliness, providing insights into the unique challenges faced by TS patients.

Moreover, the present results do not provide evidence for specific interventions targeting social isolation and loneliness in individuals with MI or TS. However, the review highlighted the mediating role played by lifestyle factors, corroborating their prognostic value. This aligns with existing evidence indicating the critical influence of lifestyle factors (e.g., tobacco use, diet, sleep, physical activity) on both physical and mental health outcomes [91]. Future studies should seek to adopt broader frameworks, testing multidisciplinary clinical programs that address not only physical activity, sleep, and diet but also subjective and interpersonal determinants of loneliness and social isolation in individuals with MI or TS. Further research is also needed to explore how psychosocial factors interact with other established risk factors (e.g., genetic predisposition, behavioral or environmental influences) for ACS. Nevertheless, the present findings underscore the importance of meaningful social connections for cardiovascular health, suggesting that understanding and addressing loneliness and social isolation in the care of ACS patients is not a peripheral concern but a critical component of a holistic, patient-centered approach—one that recognizes the interdependence of physical health, psychological well-being, and social relationships.

### Strengths and Limitations

The strengths of the present scoping review include its adherence to a comprehensive a priori protocol, integration of both quantitative and qualitative evidence, and synthesis of knowledge on subjective experiences and health outcomes. In addition, the review identified key research gaps to inform future studies. Nonetheless, several limitations should be acknowledged. First, the study design did not support the generation of clinical recommendations. However, this aligns with the study objective, which was to map the breadth and scope of the literature and indicate directions for future research. Second, the included studies were highly heterogeneous, thereby limiting the ability to draw direct comparisons across reports. Of note, this heterogeneity reflected different methodological perspectives, with qualitative studies emphasizing subjective experiences and quantitative studies focusing on physical and mental health outcomes. Third, no studies from Africa, Asia, or South America were identified, highlighting the need for cross-cultural research in this field. Finally, due to the variability in study designs, no generalizable conclusions could be drawn regarding factors influencing clinical outcomes, such as antidepressant prescriptions, cardioprotective medications, and patient frailty. Future research should address these gaps to enhance the understanding of social isolation and loneliness in cardiovascular health.

## 5. Conclusions

The present study reviewed the literature on loneliness and social isolation in individuals with MI. The findings highlight the impact of MI on subjective experiences and interpersonal relationships, often contributing to social isolation and loneliness. Moreover, both conditions were shown to be associated with adverse physical health outcomes, including increased mortality risk, with lifestyle factors playing a significant mediating role. Future research is needed to explore loneliness and social isolation in individuals with TS.

## Figures and Tables

**Table 1:** Characteristics of the included studies.

Authors, Year	Country	Sample of Interest	Focus on Social Isolation or Loneliness	Measure/Tool to Assess Social Isolation or Loneliness	Aims	Study Design
Baigi et al. [36]	Sweden	90 MI patients, representing a control condition in a study on non-attendees	Social isolation	A set of items in a specifically designed questionnaire	To examine non-attendee attitudes towards a cardiac rehabilitation program focused on risk factors and professional involvement, comparing them with those of MI attendees.	Cross-sectional
Cleophas et al. [37]	Netherlands	42 MI patients and 48 matched controls	Social isolation	A set of items in a modified version of the Ruberman questionnaire [45]	To investigate psychosocial factors, including social isolation, before, during, and after MI in Dutch men under 60 years.	Longitudinal cohort
Dickens et al. [38]	United Kingdom	314 patients with first MI	Social isolation	Frequency of social contact; socially isolated individuals had very infrequent social contact (e.g., living alone, restricted mobility)	To examine whether factors contributing to depression before and after MI were comparable to those in the general population.	Longitudinal cohort
Dreyer et al. [39]	United States	42 patients with at least one MI in the past 24 months	Social isolation	Semi-structured interview in the context of participatory action research	To understand patients’ experiences of MI and its treatment, with the aim of developing a new conceptual framework for patient-centered recovery in cardiology.	Cross-sectional
Ecochard et al. [40]	France	671 MI patients who underwent coronary angiography	Social isolation	A set of items in the Nottingham Health Profile [52]	To investigate the correlation between MI indicators assessed within the first month post-MI and perceived quality of life 1 year later.	Longitudinal cohort
Freak-Poli et al. [51]	Australia	Among 11,486 individuals, 4.2% experienced first-time cardiovascular disease (including MI) over a mean follow-up period of 4.4 years	Both social isolation and loneliness	Two questions from the Revised Lubben Social Network Scale (LSNS) [53] (social isolation) and a single item from the Center for Epidemiological Studies–Depression (CESD) scale [54] (loneliness)	To evaluate social isolation, limited social support, and loneliness as predictors of cardiovascular disease.	Longitudinal cohort
Hakulinen et al. [41]	United Kingdom	479,054 individuals, 5731 of whom had a first MI within a mean follow-up of 7.1 years	Both social isolation and loneliness	A three-item scale (social isolation) and a two-item scale (loneliness), in line with a previous UK Biobank study [55]	To examine whether social isolation and loneliness predicted MI and stroke and affected mortality risk and how these relationships were influenced by known risk factors and chronic conditions.	Longitudinal cohort
Ickovics et al. [42]	United States and Canada	2145 MI patients	Social isolation	Absence of participation in clubs/organizations, infrequent visits to friends, and limited communication with friends/family	To determine whether social class independently affected functional recovery after MI, accounting for relevant clinical, demographic, and psychosocial factors (including social isolation).	Longitudinal cohort
Liang et al. [43]	United Kingdom	19,360 individuals with type 2 diabetes mellitus, 1503 of whom had a first MI within a mean follow-up of 12.4 years	Both social isolation and loneliness	A three-item scale (social isolation), in line with previous research [55,56], and the short-term UCLA Loneliness Scale [57] (loneliness)	To examine whether social isolation and loneliness were linked to major adverse cardiovascular events (including MI), whether these associations differed between fatal and non-fatal outcomes, and how behavioral, psychological, and physiological factors mediated these relationships.	Longitudinal cohort
Liljeroos et al. [44]	Sweden	92 MI patients at the beginning of an internet-based cognitive behavioral therapy program	Both loneliness and social isolation	Evaluated through patient-written testimonies examined using qualitative content analysis	To explore patients’ emotional responses after MI and examine their self-management of emotional distress using an explanatory behavioral model.	Cross-sectional
Ruberman et al. [45]	United States	2320 MI survivors	Social isolation	One psychosocial category derived from the Health Insurance Plan BHAT questionnaire	To compare MI patients with low and high education levels regarding psychosocial characteristics and determine whether socially isolated MI patients represented a subgroup at high risk of death.	Longitudinal cohort
Sundler et al. [46]	Sweden	10 MI patients	Loneliness	Evaluated through interviews using a reflective lifeworld approach and phenomenological epistemology	To examine the significance of close relationships and sexuality for women’s health and well-being after MI.	Cross-sectional
Svedlund & Danielson [47]	Sweden	9 MI patients and their partners	Loneliness	Evaluated through interviews interpreted using a phenomenological hermeneutic method	To explore the meaning of daily life experiences following MI, as recounted by affected women and their partners.	Longitudinal cohort
Thiel et al. [48]	United States	50 MI patients and 50 age-matched healthy controls	Loneliness	Evaluated through interviews using a standardized questionnaire	To determine whether psychosocial factors, including loneliness, differed between MI patients and healthy controls	Cross-sectional
Thompson & Watson [49]	United Kingdom	668 MI patients	Loneliness	A single-item measure	To investigate the structure of the Myocardial Infarction Dimensional Assessment Scale (MIDAS) [58], designed to assess health-related quality of life in MI patients.	Cross-sectional
Zuccarella-Hackl et al. [50]	Switzerland	154 MI patients	Loneliness	The “lonely” cluster featured the lowest scores on resilience and social support, alongside average scores on task-oriented coping and positive affect	To explore the relationship between clusters of positive psychosocial factors and MI-induced depressive and post-traumatic stress symptoms.	Longitudinal cohort

Note: MI = myocardial infarction.

## Data Availability

The data that supports the findings of this study are available from the authors upon reasonable request from the corresponding authors.

## Abbreviations

The following abbreviations are used in this manuscript:

CVD	Cardiovascular disease
ACS	Acute coronary syndrome
MI	Myocardial infarction
STEMI	ST-segment elevation myocardial infarction
NSTEMI	Non-ST-segment elevation myocardial infarction
TS	Takotsubo syndrome
HPA	Hypothalamic–pituitary–adrenocortical

## Appendix A

**Table A1:** Preferred Reporting Items for Systematic reviews and Meta-Analyses extension for Scoping Reviews (PRISMA-ScR) Checklist.

Section	Item	Prisma-ScR Checklist Item	Reported on Page
**Title**			
Title	1	Identify the report as a scoping review.	Title page
**Abstract**			
Structured summary	2	Provide a structured summary that includes (as applicable) background, objectives, eligibility criteria, sources of evidence, charting methods, results, and conclusions that relate to the review questions and objectives.	Abstract
**Introduction**			
Rationale	3	Describe the rationale for the review in the context of what is already known. Explain why the review questions/objectives lend themselves to a scoping review approach.	3–4
Objectives	4	Provide an explicit statement of the questions and objectives being addressed with reference to their key elements (e.g., population or participants, concepts, and context) or other relevant key elements used to conceptualize the review questions and/or objectives.	4
**Methods**			
Protocol and registration	5	Indicate whether a review protocol exists; state if and where it can be accessed (e.g., a Web address); and, if available, provide registration information, including the registration number.	4
Eligibility criteria	6	Specify characteristics of the sources of evidence used as eligibility criteria (e.g., years considered, language, and publication status) and provide a rationale.	5
Information sources	7	Describe all information sources in the search (e.g., databases with dates of coverage and contact with authors to identify additional sources), as well as the date the most recent search was executed.	4
Search	8	Present the full electronic search strategy for at least 1 database, including any limits used, such that it could be repeated.	4
Selection of sources of evidence	9	State the process for selecting sources of evidence (i.e., screening and eligibility) included in the scoping review.	5
Data charting process	10	Describe the methods of charting data from the included sources of evidence (e.g., calibrated forms or forms that have been tested by the team before their use and whether data charting was done independently or in duplicate) and any processes for obtaining and confirming data from investigators.	5
Data items	11	List and define all variables for which data were sought and any assumptions and simplifications made.	5
Critical appraisal of individual sources of evidence	12	If done, provide a rationale for conducting a critical appraisal of included sources of evidence; describe the methods used and how this information was used in any data synthesis (if appropriate).	Not applicable
Synthesis of results	13	Describe the methods of handling and summarizing the data that were charted.	5
**Results**			
Selection of sources of evidence	14	Give numbers of sources of evidence screened, assessed for eligibility, and included in the review, with reasons for exclusions at each stage, ideally using a flow diagram.	5, Figure 1
Characteristics of sources of evidence	15	For each source of evidence, present characteristics for which data were charted and provide the citations.	5, Table 1
Critical appraisal within sources of evidence	16	If done, present data on critical appraisal of included sources of evidence (see item 12).	Not applicable
Results of individual sources of evidence	17	For each included source of evidence, present the relevant data that were charted that relate to the review questions and objectives.	8–10
Synthesis of results	18	Summarize and/or present the charting results as they relate to the review questions and objectives.	8–10
**Discussion**			
Summary of evidence	19	Summarize the main results (including an overview of concepts, themes, and types of evidence available), link to the review questions and objectives, and consider the relevance to key groups.	10–12
Limitations	20	Discuss the limitations of the scoping review process.	12–13
Conclusions	21	Provide a general interpretation of the results with respect to the review questions and objectives, as well as potential implications and/or next steps.	13
**Funding**			
Funding	22	Describe sources of funding for the included sources of evidence, as well as sources of funding for the scoping review. Describe the role of the funders of the scoping review.	13

PRISMA-ScR = Preferred Reporting Items for Systematic Reviews and Meta-Analyses extension for Scoping Reviews. Retrieved from [32].

## Appendix B

### Minor Deviations from the Original Protocol

We explored both the subjective experience and attitudes associated with loneliness/social isolation in individuals with MI or TS. Thus, we did not focus only on the subjective experience as reported in the original protocol.
